# Letters to the editor represent the apogee of peer review: A current exemplar

**DOI:** 10.1113/EP093736

**Published:** 2026-03-14

**Authors:** Timothy I. Musch, Kiana M. Schulze, David C. Poole

**Affiliations:** ^1^ Department of Anatomy and Physiology, College of Veterinary Medicine Kansas State University Manhattan Kansas USA; ^2^ School of Health Sciences, College of Health and Human Sciences Kansas State University Manhattan Kansas USA; ^3^ Translational Vascular Physiology Laboratory, Department of Internal Medicine The University of Utah Salt Lake City Utah USA

In many regards, journals that permit and encourage Letters to the Editor that critique published works are to be lauded for facilitating the highest form of peer review for scientific discovery. Rather than sole reliance on an Associate Editor and their necessarily restrictive selection of two or three ‘experts’ in the topic under consideration, Letters permit and, indeed, encourage review of a scientific paper by the entire journal readership and well beyond. They also contribute disproportionately to the journals’ impact factor by elevating the total citation count but not the denominator for the impact factor calculation, which is restricted to review and research articles (Zhang et al., 2024). From ∼2018 to 2023 there was an ∼20% increase in Letters published.

Some of the most incisive Letters challenge core mechanisms and concepts, provide methodological critiques and/or engage in philosophical debates. These letters are expertise‐driven, provide constructive criticism and extol Shakespeare's directive that ‘brevity is the soul of wit’ – or at least the basis for effective illuminative communication. In physiology, the intense exchange between Markus Amann and Samuele Marcora (Amann & Dempsey, 2008; Marcora, 2008) in *The Journal of Physiology* illustrates just how substantial the impact of Letters can be. In these and subsequent exchanges, including a spirited Point: Counterpoint series in the *Journal of Applied Physiology* in 2010, these scientists debated the role of afferent feedback from locomotor muscles and whether effort perception is peripherally or centrally generated. These Letters helped refine our understanding of fatigue and exhaustion, clarifying extant knowledge and opening fertile avenues for further research.

As mentioned above, to retain their timeliness, punchiness and economize journal space allocation, Letters are typically short (i.e., 1000 words maximum for *AJP‐Reg*) – but not always (e.g., up to 72 pages, Zhang et al., 2024) – and the number of citations permissible can be very restrictive (i.e., 10 for *AJP‐Reg*). Although original data are supposedly forbidden from inclusion in a letter or rebuttal, they can occur, and journals may also elect to allow only a single round with a rebuttal invited and penned by the authors of the original targeted paper. Thus, this well‐intentioned process can, on occasion, truncate or prohibit open discussion of original data, thereby opposing the core principals of openness and discovery which is at the heart of reputable scientific publishing.

A recent exemplar of this counterproductive occurrence unfolded recently in our own work in a preclinical model of pulmonary hypertension: a disease that has a strong female bias and high morbidity and mortality. Our Letter (Musch et al., 2025) was initiated by an original article in 2025 in *AJP‐Reg* from Long et al. (2025), evaluating the potential of nitrate supplementation (via beet root juice) to improve physiological function in pulmonary hypertension, where cardiac, metabolic and methodological information, requisite for interpreting their data, was omitted. In due course, in response to our Letter, Long et al. (2026) graciously responded with the requested materials.

Those materials successfully allayed a dominant concern, expressed by them (Long et al., 2025, p. R322) and us previously (Musch et al., 2025), that, in their monocrotaline model of pulmonary hypertension, the ‘moderate intensity’ criterion exercise bout was set at an undisclosed treadmill speed and might not have been sufficiently intense to unveil any impact of the nitrate supplementation. Specifically, they defined moderate intensity for their criterion exercise bout as 50% ${\dot{V}}_{O_{2}\max}$, which, at first glance, appears reasonable. However, the conundrum here is that, when pulmonary hypertension reduced ${\dot{V}}_{O_{2}\max}$ to 54 mL/kg/min (Long et al., 2026), 50% of this value is ∼27 mL/kg/min which is the resting metabolic rate published for rats (Brooks & White, 1978; Musch et al., 1988). Thus, absolutely no room would be left for an exercise‐induced increase in ${\dot{V}}_{O_{2}}$ as would inevitably occur in response to any treadmill walking/running.

In their rebuttal to our letter, Long et al. (2026) informed us that the ${\dot{V}}_{O_{2}}$ of that bout averaged 42 mL/kg/min for pulmonary hypertension in the placebo condition, equating to 77% rather than 50% ${\dot{V}}_{O_{2}\max}$. Completely belying the definition of moderate intensity exercise as being below the lactate threshold (rev. Poole & Jones, 2012), we were further informed that blood [lactate] exceeded 10 mM for that bout! Many investigators (e.g., Wisløff et al., 2001) consider blood [lactate] > 6 mM as indicative of a maximal exercise bout, at least in healthy rats (or humans), and we have routinely measured an average of ∼9 mM in response to severe intensity exercise in rats (e.g., Poole et al., 2000). That Long et al.’s pulmonary hypertensive rats achieved these impressively high lactate levels completely removes the concern that the exercise was not of sufficient intensity to test their hypothesis. Specifically, it reinforces the veracity of their negative findings that single‐dose nitrate supplementation did not improve cardiovascular/muscle function, and therefore would be unlikely to enhance exercise tolerance in severe pulmonary hypertension with right ventricular heart failure. Albeit in a preclinical model, we contend that this is a most valuable observation relevant to the treatment of pulmonary hypertension patients.

Now, knowing this information, a re‐evaluation of the original muscle(s) blood flow data published in their original article (Long et al., 2025) and rebuttal (Long et al., 2026) becomes warranted. For example, some of the values found in Figure 1 of their subsequent letter (Long et al., 2026, e.g., blood flows equal to ∼60 mL/min/100 g/W in the soleus, ∼58 in the rectus femoris and ∼55 in gastrocnemius) are approximately 6–7% of those previously published by this group (Long et al., 2022, p. R566, e.g., blood flows equal to ∼1000 mL/min/100 g/W in the soleus, ∼900 in the rectus femoris and ∼800 in gastrocnemius). These latter values may be among the highest muscle blood flows ever reported. Notably, the pulmonary hypertension rats from both investigations had similar ${\dot{V}}_{O_{2}\max}$ values of 53 and 54 mL/kg/min and both blood flow determinations were performed while all rats were supposedly exercising at ‘50% of their ${\dot{V}}_{O_{2}\max}$’ – though now revealed to be considerably more intense. Why there is such a disparity remains unclear at this time – but resolution of this question, we would argue, is crucial to interpretation and utility of their published data.

Also pertaining to blood flow, the box/whisker plots in their original Figure 2 (Long et al., 2025) depict that the maximum blood flow measured at rest for the placebo group was ∼140 mL/min/100 g (minimum value ∼ 25, median ∼100 mL/min 100 g). For exercise, the maximum was ∼175, minimum ∼5 and median ∼30 mL/min/100 g. Thus, half of the blood flows measured in the soleus *at rest* for the placebo group of rats were above the median (>∼100 mL/min/100 g) whereas half the blood flows measured in the soleus *during exercise* were below the median (<∼30 mL/min/100 g). These results indicate that, for some animals, exercising soleus blood flows had to be *decreasing* from rest, which contravenes many thousands of empirical observations that rhythmic exercise elevates muscle blood flow. Historically, in our laboratory (Poole et al., 2000) and others (Armstrong & Laughlin, 1983), blood flows to the soleus have been shown to increase to levels of 270–340 mL/min/100 g during treadmill running in healthy rats.

Interestingly, if Long et al.’s (2025, 2026) very low muscle blood flows are correct – and the coloured microsphere technique has been carefully and independently validated (Bernard et al., 2000) – these data are extraordinary from at least two perspectives: (1) they would suggest that, with respect to blood flow and vascular function, the predations of severe pulmonary hypertension and right ventricular heart failure are far more extreme than previously supposed based upon less pronounced pulmonary hypertension (Schulze et al., 2023, 2025) and certainly in comparison with severe left ventricular heart failure consequent to myocardial infarction (Musch & Terrell, 1992); and (2) as pulmonary hypertensive rats were able to achieve ${\dot{V}}_{O_{2}}$ values exceeding 40 mL/min/kg at these perilously low exercising muscle(s) blood flows, there must be a substantial redistribution of cardiac output (and metabolic demand) to other vascular beds and organs. The greater energetic demands and blood flows of the diaphragm and other respiratory muscles simply cannot account for the majority of this effect (Schulze et al., 2023, 2025).

We submit that the mechanistic bases for this heretofore unrecognized consequence of severe pulmonary hypertension and right ventricular heart failure is eminently worthy of focused attention and commend Long et al. (2025, 2026) for revealing this intriguing phenomenon. It is also pertinent that neither the validity and veracity of their data nor the potentially catastrophic impact of pulmonary hypertension on exercising skeletal muscle blood flows would have been highlighted without the Letter‐to‐the‐Editor review mechanism; albeit that such was sadly curtailed by journal policy in this instance. We therefore applaud the substantial increase in popularity of Letters noted by Zhang et al. (2024) and encourage this higher level of scientific scrutiny and engagement especially across the readership and preserves of *Experimental Physiology*.

## AUTHOR CONTRIBUTIONS

Timothy I. Musch and David C. Poole drafted manuscript; Timothy I. Musch, Kiana M. Schulze, and David C. Poole edited and revised manuscript. All authors have read and approved the final version of this manuscript and agree to be accountable for all aspects of the work in ensuring that questions related to the accuracy or integrity of any part of the work are appropriately investigated and resolved. All persons designated as authors qualify for authorship, and all those who qualify for authorship are listed.

## CONFLICT OF INTEREST

None declared.
